# Combination of the First-in-Class Imipridone ONC201 and Standard Anticancer Therapies as a Rational Approach for Therapeutic Benefit

**DOI:** 10.3390/cimb47090775

**Published:** 2025-09-18

**Authors:** Brahmi Shenoy, Miloni Mandani, Meena Chintamaneni, Sonal M. Manohar

**Affiliations:** 1 Department of Biological Sciences, Sunandan Divatia of School of Science, SVKM’s NMIMS (Deemed-to-Be) University, V.L. Mehta Road, Vile Parle (W), Mumbai 400056, India; 2 Department of Pharmacology, Shobhaben Pratapbhai Patel School of Pharmacy & Technology Management, SVKM’s NMIMS (Deemed-to-be) University, V.L. Mehta Road, Vile Parle (W), Mumbai 400056, India

**Keywords:** ONC201, TIC10, dordaviprone, ONC206, ONC212, imipridones, combination therapy, drug resistance, anticancer therapy

## Abstract

The development of drugs for cancer treatment faces critical challenges due to the heterogeneity in cancers, metastatic nature of the disease, lack of efficacy, toxicity, and drug resistance. This makes it quite important to understand the complexities of cancer as well as the limitations of druggable targets. ONC201 (also known as dordaviprone/TIC10/Modeyso^TM^), a first-in-class member of the imipridone family, has been shown to kill cancer cells selectively. Recently, it has received FDA approval as the first and only treatment for recurrent H3K27M-mutant diffuse midline glioma. The unique pharmacophore, favorable therapeutic index, ability to induce TRAIL and the integrated stress response (ISR), activation of natural killer cells, and ability to diffuse across the blood–brain barrier are the unique characteristics of ONC201. ONC201 has shown effectiveness against various cancers, and this has been evident in many preclinical studies. ONC201 as a single agent, although useful, has some limitations, which could be addressed by using combination strategies. ONC201 has shown synergism with other drugs, leading to greater tumor cell death or reduced tumor growth. Next-generation imipridones, viz. ONC206 and ONC212, are more potent analogs of ONC201 and exhibit similar characteristics. In this review, we discuss the therapeutic potential of ONC201 and its analogs using combination strategies across different cancers.

## 1. Introduction

ONC201 (also known as dordaviprone/TIC10/Modeyso^TM^) is a first-in-class imipridone molecule that the FDA recently approved as the first and only treatment for recurrent H3K27M-mutant diffuse midline glioma (DMG). It is a selective antagonist of GPCR Dopamine receptor 2 (DRD2) that is associated with many malignancies [1,2]. Since ONC201 was originally discovered as a TNF-Related Apoptosis-Inducing Ligand (TRAIL) agonist, it is also called TRAIL-inducing compound 10 (TIC10). It also causes dual inactivation of the AKT and ERK pathways, leading to dephosphorylation of the Forkhead box class O3a (Foxo3a) transcription factor, which increases expression of TRAIL and death receptor 5 (DR5) [3,4]. Moreover, ONC201 induces the integrated stress response (ISR), which culminates through activation of the transcription factor ATF4 and C/EBP homology protein (CHOP) and increased DR5 expression. Both of these mechanisms cumulatively lead to increased apoptosis and, hence, are attributed to the potent anti-tumorigenic activity of ONC201 [5].

Its promising pharmacokinetic properties, shorter half-life, and selective induction of apoptosis in cancer cells make ONC201 suitable for combination therapies [3]. Further, research has revealed that ONC201 can induce non-apoptotic cell death by inducing mitochondrial damage via a TRAIL-independent route [6]. The mitochondrial serine protease caseinolytic protease P (ClpP) was discovered to be the key binding target of ONC201 [7]. As ONC201 induces ClpP activity, ClpP induces the integrated stress response pathway, which leads to disruption of mitochondrial function and inhibition of cancer cell survival [8]. Numerous studies have reported that the sensitivity of tumor cells to ONC201 depends on the induction of DR5 in a manner that is influenced by ATF4 and CHOP [9].

## 2. Mechanism of Action of ONC201 as a Single Agent in Different Cancers

**Figure 1:**
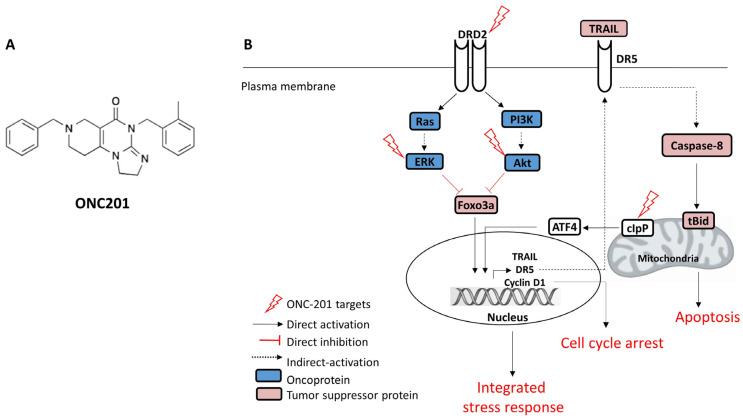
(**A**) Chemical structure of ONC201 and (**B**) basic mechanism of action of ONC201 across cancers. ONC201 antagonizes DRD2 and inhibits Akt and ERK phosphorylation, thereby restoring Foxo3a-mediated transcription of TRAIL and DR5 genes, which is further augmented by ONC201-induced ClpP and ATF4 activation. Induction of TRAIL and DR5 expression leads to enhanced extrinsic apoptosis in response to ONC201.

From several preclinical studies, it has been well established that ONC201 exhibits anti-tumorigenic properties as a single agent. The chemical structure and general mechanism of action of ONC201 across cancers are depicted in Figure 1A,B. ONC201 was originally discovered as a TNF-Related Apoptosis-Inducing Ligand (TRAIL)-inducing compound, i.e., TIC10 [9]. Subsequently, it was shown to be a DRD2 antagonist and an activator of caseinolytic protease (ClpP) [10]. It was suggested that ONC201 activates the integrated stress response (ISR) mediated by ATF4/CHOP4 downstream of DRD2 and ClpP. As per previous reports, TRAIL and DR5 levels increase in response to the ONC201-induced ISR in addition to abrogation of Akt/Erk signaling and restoration of Foxo3a-mediated transcription by this versatile drug [11,12]. Previous studies showed that ONC201 induces DR5 and TRAIL pathways without the need for p53 in cancer cells [13]. However, another study reported that ONC201 causes cell death through both TRAIL-dependent and TRAIL-independent mechanisms in breast cancer [14]. In line with these findings, one more study showed that the cytotoxicity of ONC201 is independent of both caspase cascades and death receptors in two gynecological cancers, viz. breast and endometrial cancer [15], as it was shown to induce death in these cancer cells by ATP depletion. Later, it was reported that ONC201 induces cell death via induction of cellular stress mechanisms, viz. endoplasmic reticulum (ER) stress and the atypical integrated stress response (ISR), in solid tumors as well as hematopoietic cancers [11,16,17]. Moreover, ATF4 activation may also lead to other phenotypic responses, such as TRAIL/DR5-independent apoptosis and cell cycle arrest [12]. Reportedly, ONC201-induced apoptosis via TRAIL/DR5 can be at least partially attributed to ATF4 [16]. Furthermore, ONC201 appears to have the ability to penetrate the blood–brain barrier, as demonstrated by sustained TRAIL induction in brain tissues of mice following a single dose in an orthotopic model of human GBM [3], and exert its activity against glial cancers as seen in clinical trials [18]. The ONC201-induced integrated stress response causes TRAIL-dependent apoptosis in neuroblastoma [19]. ONC201 causes decreased expression of cyclin D and pRb in endometrial cancer, and this mechanism was also shown by CDK inhibitors when used as targeted drugs in endometrial cancer [20]. ONC201 stops the growth and induces an apoptotic response in multiple cancer cell lines.

In early preclinical studies, ONC201 demonstrated single-agent efficacy in eliminating cancer stem cells in colorectal, glioblastoma, and prostate cancer models [21]. In the first in-human trial, ONC201 was shown to be well-tolerated and biologically active in advanced cancer patients upon oral administration. The pharmacokinetic profile of ONC201 at the recommended phase II dose, i.e., 625 mg, indicated rapid, significant absorption of the drug with oral administration, as indicated by a 1.8-h mean Tmax. Importantly, other pharmacokinetic parameters, viz. Cmax and AUC, in the top-dose cohort treated with the same dose exceeded those associated with the antitumor efficacy in mouse models [22]. A phase II clinical study reported on safety and suggested that this drug exerts single-agent activity in recurrent glioblastoma [23]. However, later preclinical studies showed that ONC201 exerts only an anti-proliferative response rather than a cytotoxic response in some of the cancer cell lines, mainly due to insufficient TRAIL induction [14,24,25,26,27]. ONC201 monotherapy did not demonstrate clinical efficacy in a phase II study of patients with breast or endometrial cancer [20]. Such findings prompted efforts toward developing treatment regimens that include ONC201 in combination with standard therapies for improving responses. In in vitro studies on endometrial and breast cancer cell lines, pre-treating the cells with ONC201, before TRAIL treatment, was shown to sensitize the cells toward apoptosis [28,29]. Using suboptimal doses of TRAIL with ONC201 reduces the risk of potential toxicity to the liver. In recent clinical trials of ONC201 for diffuse intrinsic pontine glioma (DIPG), patients were shown to invariably develop resistance to ONC201 mediated by PI3K/Akt hyperactivation. This prompted a combination of ONC201 with paxalisib, a PI3K inhibitor, and this combination demonstrated tumor regression and prolonged survival in diffuse intrinsic pontine glioma (DIPG) patients [30]. ONC206 and ONC212 are recent and more potent analogs of ONC201 that exhibit a similar mechanism of action.

## 3. Combination Strategies Using ONC201 and/or Its Analogs Against Various Cancers

Combination therapy is a treatment strategy that combines two or more therapeutic agents and is often superior to monotherapy for cancers. It aims to combine agents with different mechanisms of action and minimal cross-resistance in order to inhibit the emergence of broad-spectrum drug resistance [31,32]. The development of targeted anticancer drugs and their use in various combinations have broadly and substantially improved the rates and durability of response to therapy. The main aim of combination strategies is to push the resistance threshold of cancer cells, thereby converting the phenotypic response from anti-proliferative to cell death, and also to use suboptimal doses of two drugs to avoid toxicity and therapy resistance. Many solid and hematological malignancies are treated with such multidrug combinations in addition to surgery and radiation [33]. Furthermore, schedule-dependent synergism has been observed with drug combinations in clinical trials [34]. We further discuss different combination strategies employed using ONC201 and its analogs (viz., ONC206 and ONC212) to achieve the maximum therapeutic benefit in various cancers.

### 3.1. Colorectal Cancer

Colorectal cancer (CRC) has an adverse prognosis with a high mortality rate [35]. It is one of the cancers most frequently faced by both genders globally [36]. It ranks as the fourth most prevalent cancer in males and the fifth most common in females in India [37]. The mainstay of treatment for localized colorectal cancer is surgical tumor excision. Chemotherapy regimens comprise capecitabine, 5-fluorouracil, irinotecan, and oxaliplatin. In more recent times, cetuximab, panitumumab, and bevacizumab—anti-EGFR and anti-VEGF monoclonal antibodies—have been added to these chemotherapy regimens. However, due to the progression of unresectable and metastatic colorectal cancers, the overall survival rates of patients have been low. Therefore, there is a need to find effective therapeutic treatments for the purpose of enhancing the long-term survival and recovery of CRC patients.

ONC201 was found to be an intriguing targeted drug for CRC cells, especially for those that are resistant to standard therapy [4]. It was shown to induce TRAIL and subsequent cell death in numerous human CRC cell lines harboring diverse oncogenic mutations, including p53 and KRAS. ONC201 exhibits its anti-CRC activity by inducing TRAIL when administered as a single agent against HCT116 CRC xenografts [3]. ONC201 can kill tumor cells, but ineffectiveness has been observed against those cell lines that have acquired resistance. Hence, it was hypothesized that the combination of ONC201 with standard drugs would increase patient survival and improve treatment efficiency. Preclinical studies have proven the efficacy of ONC201 and bevacizumab in colorectal xenografts, wherein there was a complete reduction in tumor progression and growth [1]. Notably, it was also shown to be non-toxic. Reportedly, ONC201 downregulates cancer-stem-cell-related gene expression in solid tumors [38]. In another study, ONC201 was shown to be effective against chemotherapy (5-fluorouracil)-resistant colorectal cancer stem cells in a xenograft model [39].

ONC201 and Raw Lacquer Extract (RLE) from a deciduous tree (*Toxicodendron vernicifluum*) in combination have demonstrated an effective decrease in cell viability, proliferation, motility, and invasion in the HCT116 human colon cancer cell line [40]. This combination has been revealed to induce TRAIL activity and activation of the DR5 receptor and caspase-8. RLE has anti-inflammatory and anti-bacterial activities, and it has been proven to be beneficial to CRC patients (better overall survival and no post-treatment side effects). Fisetin is an active compound in *T. vernicifluum* that inhibits the growth of cancer cells and blocks the phosphorylation of S6K, mTOR, and AKT [41]. It has been observed that ONC201 inhibits mTOR signaling and causes apoptosis in chemoresistant CRC. This was evident in a tumor xenograft model [42].

### 3.2. Pancreatic Cancer

Pancreatic cancer is a highly chemoresistant malignancy with low survival rates [43]. It accounts for 1.03% of all malignancies; however, in India, it has significantly higher prevalence in older adults (between 65 and 75 years). This also varies by geography, with the Northeastern states having the highest incidence [44].

It is treated using surgery, chemotherapy, and radiation therapy. While surgery is the only option for localized pancreatic cancer, gemcitabine-based regimens are the primary first-line treatment for metastatic pancreatic cancer [45]. Yet, gemcitabine by itself has not considerably raised long-term survival chances; patients are unlikely to live beyond five years [46]. Gemcitabine is still an essential part of palliative care; however, intensive combination chemotherapy has demonstrated higher survival rates than gemcitabine alone in advanced disease [47]. Pancreatic cancer cells frequently display altered energy metabolism, increasing their resistance to chemotherapy. Thus, combination strategies using experimental drugs that alter metabolic pathways have been proposed to be a promising approach to overcome chemoresistance [48].

The combination of gemcitabine and nab-paclitaxel, PARP inhibitors, or immune checkpoint inhibitors is an example of effective targeted medicine [49]. Development of resistance to current chemotherapies and the lack of accurate biomarkers for early identification continue to be problems in early pancreatic cancer treatment [50].

ONC201 curbs the growth of and induces an apoptotic response in multiple pancreatic cancer cell lines. However, when ONC201 was subjected to pancreatic ductal adenocarcinoma (PDAC) cell lines, it showed a higher anti-proliferative response than an apoptotic response [27]. It was observed that there is upregulation of the DR5 receptor and low levels of anti-apoptotic proteins mediating the TRAIL-induced apoptotic response. In clinical trials, ONC201 has demonstrated anticancer activity in a variety of tumor types, but pancreatic cancer cells are largely insensitive to this drug. Pancreatic cancer cells’ resistance to ONC201 has been linked to strong IGF1-R expression. A preclinical study conducted on patient-derived pancreatic cancer cell lines found that ONC201 analogs, viz. ONC212 and ONC206, exhibit promising activity against pancreatic cancer cell lines, indicating their potential as therapeutic options. Furthermore, the combination of ONC212 with the IGF1-R inhibitor AG1024 showed promise in sensitizing ONC201-resistant cells to cell death [51]. The combination of ONC201 or ONC206 with specific small-molecule receptor tyrosine kinase (RTK) inhibitors (such as crizotinib and lapatinib) is also under consideration as an ideal treatment for pancreatic cancer cells that are resistant to other therapies. In another study, ONC201 showed synergism with the TRAIL agonist TLY012 in six pancreatic cancer cell lines of the seven cell lines tested. This combination delayed tumor growth in in vivo xenograft models of PDAC and showed minimal toxicity [52].

Over 90% of pancreatic cancers harbor a KRAS mutation, which leads to the activation of PI3K/AKT and RAS/ERK signaling pathways and, therefore, drug resistance. However, targeting both pathways with two specific inhibitors would lead to severe toxicity. Gemcitabine—the main drug approved for pancreatic cancer—is metabolically unstable and hence needs to be stabilized by a prodrug approach [53]. A lipid–gemcitabine conjugate (lipid-GEM) is more stable and enters the cells by passive diffusion. The combination of ONC201 and lipid-GEM was shown to slow down pancreatic cancer growth by simultaneous inhibition of the PI3K/AKT and Ras/ERK pathways. It reduced cell migration and caused arrest of the cell cycle at the G2 phase. Dual AKT–ERK inhibition by lipid-GEM and ONC201 reduced neoplastic growth while improving T-cell tumor surveillance as observed in a genetically engineered mouse model of PDAC with a KRAS mutation and inactive p53 [54].

Lurbinectedin is a synthetic tetrahydroisoquinoline alkaloid used in chemotherapy. Lurbinectedin binds to guanine-rich regions in the minor groove of DNA to block active transcription. As a single agent, lurbinectedin effectively kills pancreatic tumor cells and also exhibits synergism with ONC201 as well as ONC212 [55]. It was hypothesized that this combination would enhance the ability of immune cells to kill tumor cells.

### 3.3. Glioblastoma

Glioblastoma multiforme (GBM) is a fatal disease with a poor clinical prognosis. Glioblastomas are treatment-resistant, and, hence, there is a need for novel therapies [56]. The initial step in the standard treatment is surgical resection. However, radical resection of the GBM primary tumor has proven to not be a cure because of the high metastatic potential of GBM. To target any remaining tumor cells, patients usually receive radiation therapy after surgery [57]. Temozolomide is given as a chemoadjuvant therapy after radiation therapy [58]. Yet, intrinsic or acquired resistance to this drug is common due to tumor heterogeneity and continuous drug exposure. Chronic hypoxia in the tumor microenvironment further complicates treatment efforts by activating pathways that increase tumor survival and resistance to treatments [59]. Other novel treatment strategies include immunotherapies such as CAR-T cells and EGFR and VEGF inhibitors [60].

GBM metabolizes glucose for the production of energy. However, upon glucose deprivation, the tumor cells start utilizing fructose, amino acids, and fatty acids so that they can rapidly proliferate [61]. Active consumption of glucose leads to a lack of glucose in the tumor microenvironment. Glioblastoma cells exhibit an intense need for energy and biomass, which is one of the hallmarks of cancer. ONC201 shuts down the AKT and ERK signaling pathways, which are direct regulators of metabolism. In addition, ONC201 can generate a stress response, deplete cancer stem cells, and cause cell death in GBM cells. ONC201 has been shown to be effective when combined with radiation and temozolomide in a GBM mouse model, greatly lowering the tumor burden and prolonging survival [62]. ONC201 overcame therapeutic resistance to temozolomide and radiation in GBM cell lines and also slowed down the growth of cancer stem cells in therapy-resistant patient samples [21,63].

Two-deoxyglucose is an analog of glucose. When hexokinase phosphorylates this analog, 2-deoxyglucose phosphate is formed. This molecule cannot undergo further reactions, thus inhibiting glycolysis. The combination of ONC201 and 2-deoxyglucose has been tested for therapeutic potential in the U251 and A172 GBM cell lines. Metabolic reprogramming using 2-deoxyglucose along with ONC201 leads to the depletion of energy and enhanced anticancer activity [64]. Moreover, in GBM cells, ONC201 reduces Mcl-1 levels and shows synergism with Bcl-2 inhibitors (viz., ABT263 and ABT-199). Functional Bax, Bak, and NOXA were shown to be required for apoptosis induction by this synergistic combination [18].

ONC201 and its analog ONC206 have also been tested for activity in diffused midline glioma (DMG) models with a H3K27M mutation. Both of these drugs show potent antitumor activity in vitro as well as in DMG-patient-derived xenograft mice models [5,65]. ONC201 is effective in treating DMG by targeting the mitochondrial protease ClpP, with PIK3CA-mutated tumors being more sensitive to ONC201. As the PI3K–AKT pathway was shown to induce metabolic adaptation to ONC201-induced mitochondrial damage, to counteract the resistant ones, the PI3K/AKT inhibitor paxalisib was explored and their combination was found to be synergistic [66].

### 3.4. Lung Cancer

India has the fourth-highest prevalence of lung cancer in the world, with the fastest increase in rates occurring in urban areas [67]. Lung cancer is the most often diagnosed disease and the leading cause of cancer-related deaths in India [68]. Smoking, air pollution, and genetic predisposition are some of the factors that contribute to the prevalence of lung cancer in India, making it a serious public health concern.

Small-cell lung cancer (SCLC) is an advanced neuroendocrine carcinoma that contributes to around 15% of all lung cancers, primarily in smokers, and is linked to a very poor prognosis. It is an inflammatory disease with minimal treatment choices. Chemotherapy and radiotherapy are initially effective, but, eventually, treatment resistance often develops [69]. Although several treatments have received FDA approval, a dearth of relevant biomarkers hinders optimal treatment selection and analysis of synergistic effects [70].

The potency of the combination of ONC201 and lurbinectedin was examined in H1048 SCLC cells. H1048 SCLC cells co-treated with these drugs exhibited increased ATF4 and cleaved PARP and ClpP, indicating higher apoptosis. Lurbinectedin is used as a second-line therapy for recurrent or progressive diseases and is reported to have lower toxicity among patients. This molecule inhibits RNA polymerase II, a commonly hyperactive enzyme in SCLC known to promote tumor cell proliferation. Additionally, lurbinectedin causes apoptosis by attaching to a central guanine in trinucleotide triplets in the minor groove of DNA. Tumor cells experience acute DNA damage, which leads to increased production of proteins such as cleaved PARP, ATF4, phosphorylated Chk1, and γ-H2AX. Lurbinectedin is in FDA-approved clinical trials for SCLC and is being studied for potential synergy with other agents in various cancer cell lines [71]. ONC201–lurbinectidin combination therapy being effective and less harmful would be useful for treating patients who have developed resistance to standard chemotherapy.

ONC201 was proven to have a synergistic impact on patients with SCLC when combined with the traditional first-line chemotherapeutic drugs carboplatin and etoposide. ONC201, etoposide, and carboplatin were administered as a novel triple-drug combination to SCLC cell lines H1048 and H1105. This combination treatment enhanced the expression of ATF4 and cleaved PARP. The use of ONC201 in conjunction with etoposide and carboplatin offers a possible innovative triple-drug combination strategy for small-cell lung cancer [72].

Recently, a study showed that the combination of lurbinectedin with ONC201 revealed synergism in patient-derived SCLC cells in vitro, without any toxicity to normal lung epithelium [73].

More than 80% of all lung cancers are non-small-cell lung cancers (NSCLCs) [74]. NSCLC treatment usually starts with chemotherapy with or without immunotherapy or chemoradiation. Targeted therapies are preferable for tumors with certain mutations (EGFR, ALK, BRAF, etc.), as they improve overall survival (OS) and progression-free survival (PFS) [75]. PD-1 inhibitor monotherapy is the norm for patients with high PD-L1 expression. Because of the intricate interactions between genetic, epigenetic, and environmental variables, resistance to lung cancer is still a major therapy issue. Combination therapies can help resensitize resistant cancer cells by using several therapeutic drugs that target distinct pathways [76,77].

ONC201 exhibits cytotoxic and anti-proliferative effects on both established and primary NSCLC cells derived from patients while being non-toxic to normal lung epithelial cells. It triggers apoptosis in NSCLC cells by inducing TRAIL/DR5-dependent caspase-8 activation [78]. Synergy between ONC201 and taxanes (paclitaxel and docetaxel) has been demonstrated in NSCLC cell lines and in an NSCLC xenograft model [3].

### 3.5. Endometrial Cancer

India has a comparatively low prevalence of endometrial cancer when compared with Western nations. However, due to lifestyle variables and socioeconomic shifts, its prevalence is increasing [79]. Endometrial cancer is the most prevalent gynecological cancer. It is classified into two primary forms based on morphological characteristics. Type I is estrogen-dependent with an endometrioid morphology, while type II is non-estrogen-dependent with a serous papillary or clear cell morphology [80]. Molecular analysis has revealed significant heterogeneity within endometrial carcinomas (primarily adenocarcinomas) [81]. The mainstay of treatment is surgery, with adjuvant therapies such as chemotherapy, radiation, and chemoradiotherapy evaluated according to risk factors and disease stage [82]. Paclitaxel–carboplatin is still the gold standard for treating disseminated endometrial cancer.

Research has demonstrated that immune checkpoint inhibitors greatly improve progression-free survival when added to chemotherapy. The greatest advantages are seen in patients with certain genetic markers, like microsatellite instability-high (MSI-H) and mismatch repair deficiency (MMRd) [83]. The combination of immunotherapy and chemotherapy has led to a substantial evolution in the first line of treatment for endometrial cancer. With further research into new agents and targeted therapy, like mTOR inhibitors and anti-angiogenic agents, the treatment landscape for endometrial cancer is changing. It may eventually be incorporated into standard care [84].

In endometrial cancer models, ONC201 alone has demonstrated anti-tumorigenic effects, triggering the integrated stress response and causing cell death or growth arrest, suggesting its potential as a standalone treatment or in combination with other therapies for endometrial cancer [85]. ONC201 exhibited synergism with TRAIL and the DR5 agonist in the AN3CA and KLE endometrial cancer cell lines but not in HEC1A [28].

Furthermore, in endometrial cancer cell lines and mouse models, the combination of ONC206 and the PARP inhibitor olaparib was shown to exert synergistic anti-proliferative effects and enhanced apoptosis, indicating another possible therapeutic strategy for this cancer [86]. This combination was potent against both the PTEN wild-type HEC-1A and PTEN mutant ECC-1 cell lines and showed efficacy in a genetically engineered mouse model of endometrial cancer with inactive Lkb1 and p53. In phase II clinical trials, ONC201 monotherapy was found to be ineffective in recurrent or refractory metastatic endometrial cancer, but it showed an acceptable safety profile.

The mouse double minute 2 (MDM2) homolog has become a target of increasing interest as a frequently amplified marker of tumor aggressiveness across several cancers. MDM2 is an E3-ubiquitin ligase that targets p53. Milademetan (RAIN-32) is an inhibitor of the MDM2–p53 complex, which stabilizes p53 [87]. ONC201 has also been shown to notably lower detectable MDM2 levels in cell lines after treatment. An initial analysis of this impact points to synergy between milademetan and ONC201, as the increase in MDM2 levels caused by MDM2–p53 inhibition is eliminated. This combination is being investigated further and could be a unique translatable therapeutic strategy, especially when combined with checkpoint blocking [88].

### 3.6. Ovarian Cancer

In India, ovarian cancer is the third most frequent gynecological cancer in women, making it a serious health problem. Poor survival rates and a high incidence of late-stage diagnosis characterize the prevalence of ovarian cancer in India [89,90]. With a typical diagnostic age of about 50 years, the disease primarily affects women under the age of 55. The low long-term survival rates seen in the nation are partly caused by the fact that most cases are detected at advanced stages (III and IV).

With a variety of clinical traits, histological subtypes, and treatment obstacles, ovarian cancer is a difficult and aggressive illness [81]. Since ovarian cancer is asymptomatic until late stage, early detection is essential for increasing survival rates, underscoring the need for efficient biomarkers and diagnostic techniques. Cytoreductive surgery and chemotherapy are the mainstays of the first-line standard treatment for ovarian cancer. For patients with advanced-stage illness, carboplatin and paclitaxel are the usual chemotherapy regimen. Treatment responses are complicated by the different cellular features of ovarian cancer [91]. Chemotherapy resistance is a result of changes in oncogene pathways and DNA repair mechanisms [92,93]. Nearly 70% of patients relapse, suggesting that first-line treatments require ongoing innovation [94]. Recent developments have incorporated targeted therapies like poly (ADP-ribose) polymerase inhibitors (PARPis) and bevacizumab into the treatment regimen, improving overall survival (OS) and progression-free survival (PFS) rates, particularly in patients with homologous recombination deficiencies or BRCA mutations. Immune responses are hampered by complicated interactions within the tumor microenvironment and low levels of tumor-infiltrating lymphocytes (TILs) [79]. Through apoptosis induction and inhibition of pathways such as PI3K–AKT–mTOR and Ras–ERK, ONC201 has demonstrated efficacy in ovarian cancer [95].

ONC201 showed synergism with the standard therapeutic drug paclitaxel in high-grade and low-grade ovarian cancer cell lines, irrespective of their platinum sensitivity [96]. Additionally, in high-grade serous ovarian cancer cell lines, the combination of ONC201 and ceralasertib (an AKT inhibitor) has shown synergistic effects, improving cell viability reduction [97]. Furthermore, ONC201 exhibited a synergistic response in combination with the Bcl-2/Bcl-xl inhibitor navitoclax (ABT-263) by altering the balance between pro-apoptotic (NOXA, Bax) and anti-apoptotic (Mcl-1, BAG3) mediators [98]. All these studies suggest possible therapeutic strategies for the treatment of this aggressive type of cancer. A combination of ONC201 and paclitaxel is being tested in phase II trials for the treatment of platinum-resistant ovarian cancers [99].

### 3.7. Triple-Negative Breast Cancer

Triple-negative breast cancer (TNBC) is common in India, with an incidence rate of about 20–30% [100]. Because of its poor prognosis and limited treatment options, this aggressive subtype of breast cancer presents substantial hurdles in clinical management [101]. According to a comprehensive review, about 30% of people have TNBC. Although 55 is the median age of TNBC patients in India, younger women make up a sizable fraction of this population [102].

For the majority of TNBC cases, preoperative or neoadjuvant chemotherapy, which may include targeted medications, has become the accepted course of treatment [103]. When compared with conventional chemotherapy alone, this method improves overall survival (OS) and progression-free survival (PFS) [104]. Because of its aggressiveness and genomic heterogeneity, triple-negative breast cancer (TNBC) presents a serious challenge in terms of treatment resistance [105,106].

According to research, ONC201 causes TRAIL expression, which can impact both TNBC and non-TNBC cells and, in certain situations, result in cell death [107]. Moreover, ONC201 binds to and activates mitochondrial ClpP, which affects breast ductal carcinoma cells by causing a stress response and reversible growth arrest [108]. Prolonged exposure results in responses such as cell cycle arrest and inhibition of cell proliferation [109].

Studies show that everolimus plus ONC201/TIC10 is effective in therapy-resistant ER+ breast cancer cells [110]. ONC201’s anti-proliferative activity in everolimus-resistant cells has been proposed to be via c-Myc inhibition. Furthermore, combining ONC201 and the Bcl-2 inhibitor ABT-263 appears to have substantial synergistic effects that cause tumor cell death according to a study on solid tumor cells from a variety of organs, including the breast [20].

A study demonstrated the synergistic action of a combination of rucaparib (a PARP inhibitor) and imipridone (ONC212/ONC201) in BRCA1/2-deficient breast, ovarian, and prostate cancer cell lines [111]. The dual-drug combination showed enhanced ATF4 and reduced total Akt levels. Testing ONC201 with trametinib (an MEK inhibitor) in ONC201-sensitive (CAL51) and ONC201-resistant (HCC70) TNBC cells demonstrated their synergistic effectiveness in an ex vivo assay [112]. However, the authors of the study could not decipher the mechanism underlying this synergy beyond caspase-3/7-mediated apoptosis induction. These results demonstrate the potential of ONC201 and its combinations as potent therapeutic approaches to breast cancer treatment.

### 3.8. Prostate Cancer

According to several reports, the incidence of prostate cancer has been increasing alarmingly in India [113,114]. About 43% of patients are diagnosed at a metastatic stage. The primary focus of first-line treatments is hormone therapy, specifically androgen deprivation therapy (ADT), which has been the mainstay of treatment for more advanced patients [115]. Docetaxel is a cytotoxic drug that is frequently used as a first-line treatment to improve quality of life and survival in patients with metastatic castration-resistant prostate cancer (mCRPC). The tumor microenvironment contributes significantly to therapeutic resistance [116]. There are many pathways that contribute to this resistant phenotype, such as the PI3K–Akt–mTOR pathway and the EGFR signaling pathway [117].

ONC201 is seen as an efficacious treatment for prostate cancer in preclinical studies. ONC201 showed a clinical benefit in prostate cancer patients during early clinical trials [22]. It exhibited anti-proliferative and apoptotic effects in prostate cancer cells by downregulating the master drivers AR-V7 (an activated AR variant that lacks the C-terminal binding domain) and PSA (a gene targeted by AR) in both castration-resistant and sensitive prostate cancers when tested in vitro [25].

Many studies have explored the potential of ONC201 in LNCaP prostate cancer xenograft models as single agent. The combination of ONC201, darolutamide, and enzalutamide worked synergistically to reduce cellular viability, induce apoptosis, and downregulate AR signaling in a castration-resistant prostate cancer model [118]. Also, the combination of ONC201 and enzalutamide inhibits the growth of prostate cancer stem cells. The elevation in intratumor NK cells, TRAIL activity, and activation of TRAIL cause tumor cell apoptosis upon treatment with ONC201, also triggering the integrated stress response (ISR) [119].

In castration-resistant prostate cancer, ONC201/TIC10 in combination with enzalutamide and darolutamide exhibits promising antitumor activity by targeting androgen receptor signaling and integrated stress response pathways [118]. Ceralasertib is a strong and specific orally bioavailable inhibitor of the ATR kinase. When used as a monotherapy, it has a modest anticancer effect against prostate cancer. Combination therapy with ceralasertib and ONC201 demonstrated a synergistic response in prostate cancer cell lines, as ONC201 induced TRAIL-dependent apoptosis and the integrated stress response [120].

As mentioned above, breast, ovarian, and prostate malignancies harbor BRCA1/2 mutations. Tumor cells with BRCA1/2 mutations are susceptible to PARP inhibitors (PARPis), which disrupt the DNA replication fork. Studies have postulated that imipridones, when combined with a PARPi (such as olaparib or rucaparib), would overcome PARPi resistance (which is mediated by hyperactivation of AKT). Additionally, PARPis make different solid tumors more sensitive to recombinant TRAIL and DR5 agonists/antibodies, as imipridones and PARPis work in concert. In BRCA-deficient cancer cells, the effectiveness of the imipridone ONC201 as a single agent and its synergy in combination with olaparib have been demonstrated. Thus, the combination of an imipridone (ONC212/ONC201) and rucaparib (a PARP inhibitor) in BRCA1/2-deficient breast and ovarian cancer cell lines offers therapeutic promise [111]. Synergy was also observed for such a combination in the case of prostate cancer and glioblastoma cells [121].

### 3.9. Gastric Adenocarcinoma

Gastric adenocarcinoma, which has two different histological subtypes (diffuse and intestinal), is a serious global health concern. Localized disease is treated with amputation, whereas advanced stages require chemotherapy and/or radiotherapy [122]. Molecular assessments illustrate genetic variations among different subtypes that influence prognosis [123]. The most widely used treatment regimens are DCF (Docetaxel, Cisplatin, and 5-Fluorouracil) and FLOT (Fluorouracil, Leucovorin, Oxaliplatin, and Docetaxel), which have demonstrated encouraging outcomes in terms of progression-free survival and overall survival [124]. Personalised approaches are necessary due to the treatment resistance caused by the distinct genetic features of these tumors [125].

In order to determine the exact mechanism of action of ONC201, researchers have looked into its capacity to cause cell death in gastric adenocarcinoma cells when combined with recombinant human TRAIL (rhTRAIL) as well as its impact on the expression of inhibitor of apoptosis (IAP) and DR5. Dual therapy with ONC201 and rhTRAIL resulted in substantial synergy across all cell lines, with combination indices < 0.6 at doses that did not cause cell death in normal fibroblasts. After administering ONC201 and rhTRAIL in combination, the results proved that apoptosis was due to enhanced fragmentation of PARP, caspase-8, and caspase-3 as compared with rhTRAIL alone. ONC201 led to downregulation of anti-apoptotic proteins, viz. cIAP-2 and XIAP, and upregulation of ATF4 and CHOP, indicating activation of the integrated stress response pathway. ONC201 in combination with rhTRAIL also exhibited ISR activation, enhanced DR5 cell surface expression, and downregulation of apoptosis inhibitors [126]. This proved that this combination may be a viable and non-toxic approach for the treatment of gastric cancer. This combination is now being explored utilizing AGS and SNU-1 cells in an organoid model and a mouse subcutaneous xenograft model. ONC201 in combination with a recently developed PEGylated trimeric TRAIL formulation (TLY012) showed antitumor effects in vivo against gastric cancer, and this combination may be further explored in clinical trials [127].

**Figure 2:**
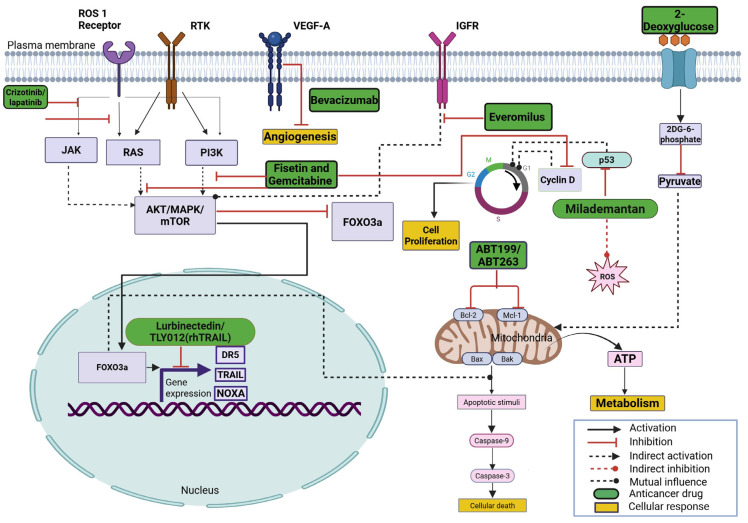
Molecular targets of various anticancer drugs that exhibit synergy in combination with imipridones (ONC201, ONC206, and ONC212).

**Figure 3:**
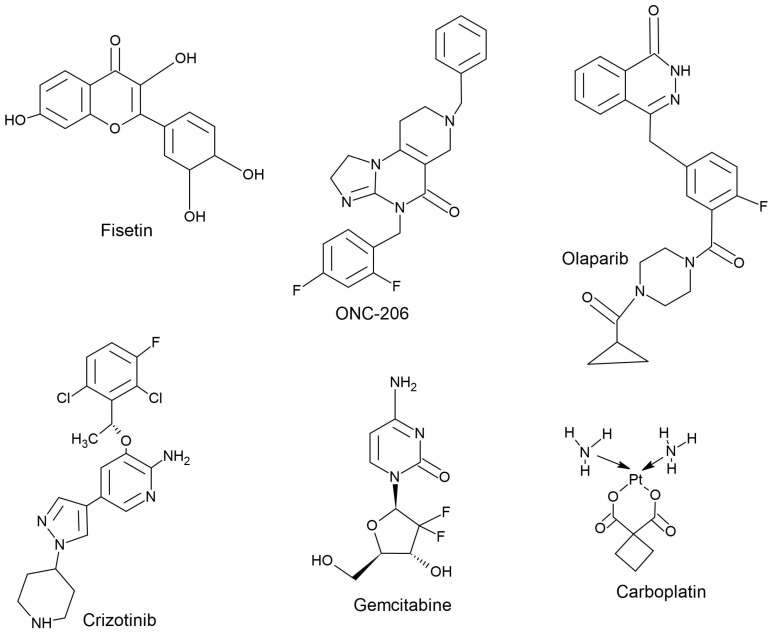
Chemical structures of ONC206 (an ONC201 analog) and standard anticancer drugs that have demonstrated synergism in combination with ONC201.

Table 1 summarizes preclinical studies of combinations of imipridones (i.e., ONC201, ONC206, and ONC212) with various anticancer drugs in different types of cancers. A schematic of the mechanism of action of these anticancer drugs is shown in Figure 2, and representative chemical structures are shown in Figure 3.

## 4. Limitations of/Current Challenges with ONC201-Based Anticancer Therapy

ONC201, as a dual AKT and ERK inhibitor, has shown promise in preclinical models and is now the first and only drug approved by the FDA for the treatment of recurrent pediatric and adult H3K27M-mutant diffuse midline glioma (DMG). The safety features of ONC201/TIC10 that were key selection criteria offer a range of clinical opportunities where genotoxic and toxic therapies are intractable [133]. Furthermore, combination therapy may be facilitated by the absence of overlapping toxicities and the broad synergy with approved anticancer compounds that has recently been reported for ONC201/TIC10. Exploration of small molecules that dually inhibit the PI3K–AKT and MAPK–ERK pathways may represent a novel practical strategy in the development of new anticancer agents. It is worth keeping in mind the heterogeneity and complexity of the signaling networks in cancer cells. The PI3K–AKT and MAPK–ERK pathways are important oncogenic cascades and play a critical role in cancer development and progression, but other oncogenic pathways, such as WNT signaling in colorectal cancer, breast cancer, and hepatocellular cancer, may be intrinsically activated or induced in response to inhibition of the PI3K–AKT and MAPK–ERK pathways. Therefore, even promising dual AKT and ERK inhibitors may face unexpected limits in clinical practice [134]. Also, although imipridones suppress the main energetic metabolic pathways of GBM cells, compensatory pathways have been shown to be activated mainly by ATF4-induced transcriptional changes. These pathways are crucial for the survival of GBM cells as well as colon cancer cells [130].

Although caseinolytic protease (CIpP) has been identified as a direct binding target of ONC201, it seems to act independently of CIpP via different mechanisms in diverse types of cancers [17,26]. It is imperative to understand these mechanisms for the effective clinical development of this targeted drug. In addition, predictive biomarkers that identify sensitive and resistant cells must be identified so as to carefully select patient cohorts that are most likely to benefit [135]. The feasibility of using cerebrospinal fluid (CSF) H3K27M-DMG cell-free tumor DNA (tDNA) as a biomarker of ONC201 response was recently demonstrated [136]. It has also been suggested that early reversal of H3K27 trimethylation with ONC201 treatment represents a potential prognostic biomarker that can be assessed in future clinical trials using target validation [65]. Genomics of Drug Sensitivity in Cancer (GDSC) analyses in a large panel of cancer cell lines suggested that a single biomarker may not completely capture the sensitivity profiles of ONC201 and ONC206 across tumor types and that combinations of biomarkers would increase the predictive power. Hypoxia-related gene expression analysis indicated that hypoxic tumor cells could be resistant to ONC201 and ONC206 [137]. EGFR mutations, EGFR overexpression, and MAPK pathway alterations have been shown to be markers of resistance to ONC201 among H3K27-DMG patients in phase II clinical trials [138]. Cleaved caspase-3 and Ki67 have recently been proposed as short-term pharmacodynamic biomarkers for the combination of ONC201, temozolomide, and radiation based on in vivo studies using an orthotopic GBM mouse model [131]. Apart from this, Bonner et al. [139] have proposed a list of candidate biomarkers for sensitivity to ONC201 and other imipridones (Bim, ClpP, c-Myc, DRD2, GPR132, mTOR, IGF1R, and XIAP are some of these candidate biomarkers). Additionally, monitoring of NK cell numbers and NK infiltration of tumors post-treatment has been suggested to be useful for understanding the clinical efficacy of ONC201 [119].

In the past, there was a discrepancy between the reported structure of ONC201 (TIC10) (with a linear inactive core) and the active structure (with an angular active core), which was confirmed by extensive analysis using NMR and X-ray crystallography [135]. Thus, close attention to the chemical structure of TIC10/ONC201 used in any further studies is crucial for preclinical and clinical development.

## 5. ONC201 Analogs

Based on the favorable pharmacological properties of ONC201, such as its safety, pharmacokinetics, pharmacodynamics, and bioavailability, a structure–activity relationship (SAR) approach was used to enhance the anticancer efficacy of its novel, tri-heterocyclic scaffold. The SAR approach led to the identification of new imipridones that retain the core structure of ONC201 while exhibiting engagement with ONC201 targets, such as DRD2 and ClpP. ONC206 and ONC212 are fluorinated ONC201 derivatives with nanomolar potency. Like ONC201, their antitumor effect also involves hyperactivation of ClpP, initiating tumor-specific apoptosis. Thus, there is interest in further evaluation of imipridone analogs such as ONC206 and ONC212 in GBM and DIPG [140]. ONC206 and ONC212 display higher potency against tumor cells, including GBM (15, 16). ONC206 has entered a phase I trial for solid central nervous system malignancies, including glioblastoma and gliosarcoma (NCT04541082) [141]. ONC206 was shown to be 10 times more potent against diffuse midline glioma (DMG) than ONC201 [142,143]. Treatment of DMG with an imiprodone triggered a lineage shift from a proliferative, oligodendrocyte-precursor-like state to a mature, astrocyte-like state in tumor cells. ONC201/ONC206 + RT/TMZ (IRT) therapy reduced the intracranial tumor burden, prolonged survival in an orthotopic IDH-WT GBM mouse model and suppressed MGMT [131]. The imipridones ONC201 and ONC212 have been proposed to be potent OXPHOS inhibitors for targeting leukemic stem cells in acute myeloid leukemia [144].

Furthermore, ONC206 has demonstrated anti-tumorigenic activity in endometrial cancer and ovarian cancer [145,146,147] and has also exhibited synergism with olaparib in endometrial cancer [86]. ONC201 and ONC206 showed potent activity against the MYCN-amplified IMR-32 and non-MYCN-amplified SK-N-SH human neuroblastoma cell lines via a novel mechanism (inhibition of EGF-induced L1CAM and PDGFRb phosphorylation) [148]. Combination therapy with ONC201 or ONC206 and enzalutamide or darolutamide was shown to be synergistic in preclinical studies of castration-resistant prostate cancer [149]. The neuroendocrine prostate cancer drivers SOX2 and BRN2 confer differential responses to the imipridones ONC201, ONC206, and ONC212 in prostate cancer cell lines [150]. The combination of ONC201, ONC206, or ONC212 and dual EZH1–2i was found to be synergistic. We observed synergies with imipridones combined with HDACi and a combination of ONC201/ONC206, EZH2i, and HDACi in DG, GBM, prostate cancer, and SCLC cells [151]. Recently, a combination of supercharged natural killer cells with ONC201/ONC206 showed enhanced potency against H3K27 mutant gliomas [152]. Nonetheless, dopamine pre-treatment impairs the anticancer effect of ISR- and TRAIL-pathway-inducing ONC201, ONC206, and ONC212 in pancreatic and colorectal cancer, but not DMG cells [153]. This hints toward the higher potency of imipridones toward gliomas as compared with other malignancies.

ONC212 shows enhanced apoptotic-inducing potential when combined with phosphoglycerate dehydrogenase (PDGDH) inhibitors against GBM cells and reduced tumor size in GBM and colon carcinoma models [130]. ONC212 also exhibited synergism with ABT-199 (a Bcl-2 inhibitor) against acute myeloid leukemia cells in vitro and in vivo [154]. When ONC201/ONC212 was combined with AG1024 (an IGF1-R inhibitor), this combination exhibited synergism in in vitro and in vivo models of pancreatic cancer [51].

## 6. Future Perspectives

ONC201 is an imipridone with proven efficacy in preclinical models of several cancers. It has shown clinical activity in tumor types that overexpress DRD2 and/or ClpP, such as certain types of high-grade glioma, endometrial cancer, prostate cancer, and mantle cell lymphoma. Further, it shows synergism with radiotherapy, chemotherapy, targeted therapy, and immunotherapy. It is the first FDA-approved systemic therapy for H3K27M-mutant diffuse midline glioma. Novel, more potent analogs of ONC201 have been developed based on SAR-based screens, viz. ONC206, ONC212, TBP-134, and PBP-135, which also exhibit potency and synergism with the standard therapies [155,156,157,158]. ONC206 is undergoing phase I clinical trials as a single agent and in combination with radiation for DMG and other recurrent primary brain tumors. ONC206 shows excellent stability in solution and human plasma [159]. Novel ferrocene–imipridone conjugates have been designed and synthesized in order to improve the efficacy of imipridones [160]. Thus, imipridones represent a useful approach to target resistant cancer stem cells as well as other cell types within the tumor microenvironment for mitigating drug resistance and enhancing therapeutic efficacy. Further, the unique mechanisms of action of these novel drugs across cancers need to be delineated for potential biomarker discovery.

## 7. Conclusions

ONC201 is a first-in-class, orally efficacious small molecule of the imipridone family that specifically kills various types of cancer cells and is currently in clinical trials for different types of cancers. Combination therapy employs suboptimal doses of two or more drugs with non-overlapping targets and toxicities, thus maximizing the efficacy while overcoming resistance to monotherapy. In the past few years, various preclinical studies have established that ONC201 and its analogs, ONC206 and ONC212, exhibit synergism with standard-of-care chemotherapy drugs across different types of cancers. The synergy observed in preclinical studies, good therapeutic index, and lack of toxicity as a single agent make imipridones ideal candidates for combination therapy so as to overcome therapy resistance in various cancers. Such treatment regimens are worth exploring in the clinic for all types of cancers.

## Figures and Tables

**Table 1:** Preclinical studies of combinations of imipridones (i.e., ONC201, ONC206, and ONC212) with various anticancer drugs in different types of cancers.

Tumor Type	Drug Used in Combination with ONC201	Cell Lines/Xenograft Models Used	References
Colorectal cancer	VEGF inhibitors like bevacizumab (Avastin) with ONC201	Mice harboring human xenografts of CRC	[128]
		CRC xenograft and patient-derived xenograft (PDX)	[1]
	Fisetin (an active compound in *T. vernicifllum*) with ONC201	HCT116 human colon cancer cell line	[40]
	mTOR inhibitors with ONC201	HT-29, HCT116, and DLD-1 CRC cell lines, chemoresistant CRC xenograft model	[42]
Pancreatic cancer	Lurbinectedin/irinotecan with ONC201/ONC212	PANC-1, BxPC-3, and HPAF-II cell lines	[55]
	TLY012 (TRAIL mimetic)	AsPC-1, BxPC3, Capan-1, and PANC-1 cell lines, patient-derived xenograft (PDX)	[52,129]
	The IGF-1R inhibitor (insulin-like growth factor) AG1024 with ONC201/ONC212	AsPC-1, BxPC3, Capan-1, Capan-2, CFPAC-1, PANC-1, HPAF-II	[51]
	Crizotinib or lapatinib with ONC201/ONC212	PANC-1, BxPC3, Capan-2, and HPAF-II cell lines and xenograft model	[51]
	Lipid–gemcitabine with ONC201	MIA-PaCa-2, syngeneic Kras-mutated pancreatic cancer xenograft mouse model	[54]
Glioblastoma (GBM)	BRD4 antagonist with ONC201/ONC206/ONC212	NCH421k, NCH644, NCH690, SF188 < LN229, U87, T98G, and GBM14 GBM cell lines, patient-derived xenograft models of GBM	[130]
	Bcl-2 inhibitors	Patient-derived glioblastoma cells, SF188 (pediatric), T98G (adult), and MGPP-3 (murine, transgenically derived) glioblastoma cells	[18]
	Temozolomide and radiotherapy with ONC201/ONC206	SNB19, T98G, U138, U251 glioblastoma cell lines, the SF8628 diffuse intrinsic pontine glioma (DIPG) cell line, orthotopic model of GBM in mice	[131]
Lung cancer	Lurbinectedin with ONC201	H1048, H1105, H1882, and H1417 SCLC cell lines	[132]
	Etoposide and carboplatin with ONC201	H1048 and H1105 SCLC cell lines	[72]
	Paclitaxel or docetaxel with ONC201	H460 human non-small-cell lung cancer xenografts in athymic nude mice	[3]
Endometrial cancer	TRAIL with ONC201	AN3CA, HEC1A, and KLE endometrial cancer cell lines	[85]
	The MDM2 inhibitor milademetan with ONC201	-	[88]
Ovarian cancer	The ATR kinase inhibitor ceralasertib with ONC201	OVCAR3, KURAMOCHI, TOV21G high-grade serous ovarian cancer cell lines	[97]
	The PARP inhibitor olaparib or rucaparib with ONC201/ONC212	HCC1937, PEO1, KURAMOCHI, 22RV1, LNCAP (BRCA-deficient cell lines of breast, ovarian, and prostate cancer)	[111]
Breast cancer (Triple-negative and ER+)	Everolimus with ONC201	MCF7, T47D ER+ breast cancer cell lines, cell lines derived from patients sensitive or resistant to everolimus	[110]
	The MEK inhibitor trametinib with ONC201	BT-20, HCC38, HCC70, HCC1187, HCC1395, HCC1806, HCC1937, MDA-MB-157, MDA-MB-231, MDA-MB-453, and MDA-MB-468 TNBC cell lines	[112]
Prostate cancer	Darolutamide or enzalutamide with ONC201	22RV1 and LNCaP cell lines, mouse xenograft models with luciferase expressing the 22RV1 and LNCaP cell lines	[118]
	The ATR kinase inhibitor ceralasertib with ONC201	-	[120]
Gastric adenocarcinoma	Recombinant TRAIL (rhTRAIL) with ONC201	AGS, SNU-1, SNU-5, and SNU-16 cell lines AGS and SNU-1 cells in an organoid model and a mouse subcutaneous xenograft model	[126]

## Data Availability

Not applicable.
